# Efficacy of *Lactobacillus paracasei* HA-196 and *Bifidobacterium longum* R0175 in Alleviating Symptoms of Irritable Bowel Syndrome (IBS): A Randomized, Placebo-Controlled Study

**DOI:** 10.3390/nu12041159

**Published:** 2020-04-21

**Authors:** Erin D. Lewis, Joseph M. Antony, David C. Crowley, Amanda Piano, Renu Bhardwaj, Thomas A. Tompkins, Malkanthi Evans

**Affiliations:** 1 KGK Science Inc., London, ON N6A 5R8, Canada; elewis@kgkscience.com (E.D.L.); josephtheval@gmail.com (J.M.A.); dcrowley@kgkscience.com (D.C.C.); 2 Lallemand Health Solutions, Montreal, QC H4P 2R2, Canada; apiano@lallemand.com (A.P.); rbhardwaj@lallemand.com (R.B.); ttompkins@lallemand.com (T.A.T.)

**Keywords:** probiotics, *Bifidobacterium longum*, *Lactobacillus paracasei*, Irritable Bowel Syndrome, IBS

## Abstract

Specific probiotic strains can alleviate the gastrointestinal (GI) symptoms and psychiatric comorbidities of irritable bowel syndrome (IBS). In this randomized, double-blind, placebo-controlled study, the efficacy of *Lactobacillus paracasei* HA-196 (*L. paracasei*) and *Bifidobacterium longum* R0175 (*B. longum*) in reducing the GI and psychological symptoms of IBS was evaluated in 251 adults with either constipation (IBS-C), diarrhea (IBS-D), or mixed-pattern (IBS-M). Following a 2-week run-in period, participants were randomized to one of three interventions: *L. paracasei* (*n* = 84), *B. longum* (*n* = 83) or placebo (*n* = 81). IBS symptoms, stool frequency and consistency and quality of life were assessed by questionnaires. The differences from baseline in the severity of IBS symptoms at 4 and 8 weeks were similar between groups. Participants in this study were classified, after randomization, into subtypes according to Rome III. Within the *L. paracasei* group, complete spontaneous and spontaneous bowel movement frequency increased in participants with IBS-C (*n* = 10) after 8 weeks of supplementation (both *p* < 0.05) and decreased in participants with IBS-D (*n* = 10, *p* = 0.013). Both *L. paracasei* and *B. longum* supplementation improved the quality of life in emotional well-being and social functioning compared with baseline (all *p* < 0.05). In conclusion, *L. paracasei* and *B. longum* may reduce GI symptom severity and improve the psychological well-being of individuals with certain IBS subtypes.

## 1. Introduction

Irritable bowel syndrome (IBS) is one of the most common functional gastrointestinal disorders (GI) worldwide, affecting approximately 11% of the global population [1]. IBS is characterized by the co-occurrence of abdominal pain or discomfort, changes in bowel habits, and defecation [2]. Based on bowel habits and stool consistency, IBS patients are grouped into three subtypes, namely the constipation-predominant IBS (IBS-C), diarrhea-predominant IBS (IBS-D), and the mixed bowel habits or cyclic patterns IBS (IBS-M) characterized by the occurrence of both constipation and diarrhea episodes [2]. The physical symptoms of IBS are variable in severity, ranging from unpleasant to debilitating, and as a result, patients’ psychological well-being and quality of life can be adversely affected in more severe cases. This variability in the severity of IBS symptoms, coupled with our incomplete understanding of its pathophysiology, often results in extended delays for patients before receiving a diagnosis. This also leads to inconsistencies in the efficacy of symptom management regimes. Currently, the first step in IBS symptom management involves lifestyle and dietary modifications, such as the adoption of low fermentable oligo-, di-, and monosaccharides and polyols (FODMAPs) and gluten-free diets [3]. In cases where dietary changes are ineffective, adjunctive pharmacotherapies such as antidiarrheal agents, antibiotics, laxatives, antispasmodics, or antidepressants are introduced. Pharmacological interventions often target specific but not all symptoms of IBS and may result in serious adverse events [4,5]. Based on accumulating research evidence, gut microbial dysbiosis is thought to play a role in the pathophysiology of IBS and symptom severity. Consequently, promising leads in IBS management now include the potential use of specific probiotic bacteria to improve both the GI symptoms and psychological comorbidities in IBS [6].

The potential use of probiotics in IBS management stems from their documented beneficial effects on GI health as well as their strong history of safe use [7]. In addition, a lower abundance of *Bifidobacterium* and *Lactobacillus* species has been observed in IBS patients [8], and a reduction in IBS symptom severity following supplementation with probiotic strains from these genera was reported by several randomized controlled trials (RCTs) [9,10]. For instance, *Bifidobacterium lactis* DN-173 010, *Lactobacillus paracasei* NCC2461, *Lactobacillus acidophilus* NCFM, *Bifidobacterium infantis* 35624, and *Bifidobacterium longum* NCC3001 have shown some efficacy at reducing GI symptoms of IBS, such as frequency of bowel movements (BMs), pain, and visceral hypersensitivity [11]. Supplementation for 2 months with *B. longum* W11 in combination with rifaximine, a broad-spectrum antibiotic, significantly reduced symptoms of IBS compared to rifaximine alone, as measured by a visual analogue scale [12]. A clinical study exploring the effects of *Lactobacillus paracasei* F19 on IBS symptoms reported an improvement in the bowel habits of participants with constipation or diarrhea and showed significant improvements in the frequency and intensity of self-reported pain [13].

Probiotics were found to exert both strain-specific and dose-dependent effects on gut health [10,14], but these effects are poorly characterized in IBS patients. Hence, characterizing the specific and dose-related effects of single probiotic strains in clinical trials could foster the development of more efficient probiotic blends tailored to IBS patients’ needs. Supplementation with *Lactobacillus helveticus* R0052 and *Bifidobacterium longum* R0175 has shown beneficial psychological effects in healthy human volunteers [15] and patients with major depressive disorder [16]. However, *B. longum* R0175 supplementation has not been studied in IBS patients. Similarly, there are no previous studies on *Lactobacillus paracasei* HA-196 in a population with IBS.

Therefore, the aim of the current study was to evaluate the efficacy of two single strain probiotics, *B. longum* R0175 (*B. longum*) and *L. paracasei* HA-196 (*L. paracasei*), in the management of GI and psychological symptoms in IBS patients.

## 2. Materials and Methods

### 2.1. Study Design

This study was approved by the Natural and Non-Prescription Health Products Directorate, Health Canada, Ottawa, Ontario on 23 June 2014. Research ethics board approval was granted on 7 July 2014 from the Institutional Review Board (IRB) Services, Aurora, Ontario. All participants in the study provided written informed consent at the screening visit and the study was conducted in accordance with the Declaration of Helsinki guidelines and its subsequent amendments. The trial was registered at ClinicalTrials.gov (NCT02213172) and followed the CONSORT guidelines for randomized controlled trials [17] (Supplementary Table S1). The study was planned to only include participants with IBS-C. Due to low enrollment, the protocol was modified on 6 January 2016 to include participants with all IBS subtypes.

**Figure 1:**
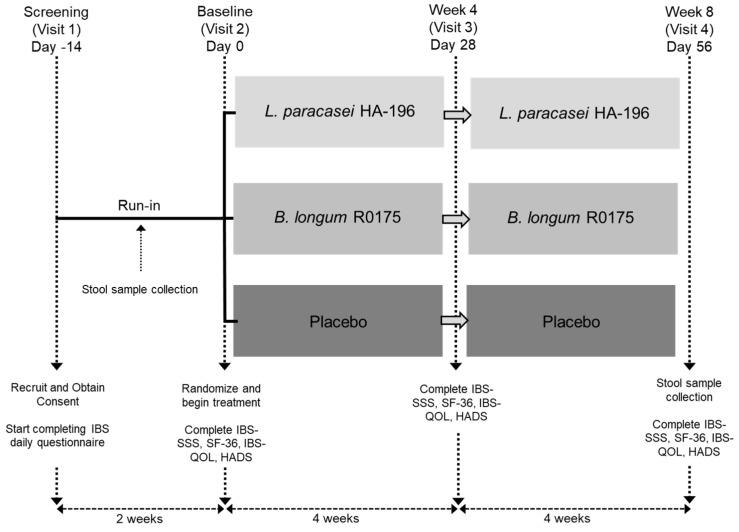
Schematic representation of the study design. This 10-week study consisted of 2 periods: a 2-week run-in and an 8-week supplementation period with one of two probiotics, *Lactobacillus paracasei* (*L. paracasei*) HA-196 or *Bifidobacterium longum* (*B. longum*) R0175 or a placebo (days 1–56).

This randomized, double-blind, placebo-controlled, 3-arm parallel group study was conducted at KGK Science Inc., London, ON, Canada between September 2014 and February 2018. The study included a 2-week run-in period, during which regular bowel habits were reported, and an 8-week intervention period (day 1 to day 57), consisting of a total of four clinic visits (Figure 1).

Clinical and qualitative assessments were conducted at screening, day 0 (baseline), 4 weeks and 12 weeks (end-of-study). At the screening visit, participants were provided with a 3-day food record and a 2-week daily diary to complete before the baseline visit. The daily diary assessed the number of spontaneous bowel movements (SBM; defined as a stool not induced by rescue medication), complete spontaneous bowel movements (CSBM; defined as an SBM associated with a sensation of complete evacuation), and stool consistency based on the Bristol stool scale [18]. Participants were also instructed to collect a fecal sample (5–10 g) using the provided stool collection kits 3 days before taking the investigational product (IP). The fecal sample was to be frozen immediately upon collection and brought to the clinic before or at the baseline visit.

At all study visits, the Irritable Bowel Syndrome Symptom Severity Scale (IBS-SSS), Short Form (36) Health Survey v2^TM^ (SF-36), Irritable Bowel Syndrome Quality of life (IBS-QOL), and Hospital Anxiety Depression Scale (HADS) questionnaires were administered and assessed. Additionally, 3-day food records and diaries were reviewed for daily IBS-SSS, concomitant therapies, adverse events, and study product use. Vital signs and anthropometric measurements were undertaken at each visit while hematology and clinical chemistry parameters for all safety endpoints were assessed at the screening and end-of-study visits.

At week 8, participants provided a fecal sample (5–10 g) collected within the 3 days preceding their last visit and frozen immediately after collection and then brought to the clinic.

### 2.2. Participants

Individuals diagnosed with IBS were recruited from Southwestern Ontario, Canada using the KGK Science Inc.’s internal participant database along with local electronic and physical advertisement devoid of gender or racial bias. Participants met the following inclusion criteria: aged 18 years or older, IBS diagnosed according to the Rome III criteria [19], and willingness to discontinue probiotic consumption for the duration of the study. After the broadening of eligibility criteria to all subtypes, the distribution of IBS subtypes in each intervention group was determined after randomization using the IBS Rome III Questionnaire completed at screening.

Participants were excluded if they used medications to manage IBS symptoms or narcotics in the past month, history of gastrointestinal surgery, gastrointestinal disease (except hemorrhoids and uncomplicated diverticula) or family history of colorectal cancer, inflammatory bowel disease, or celiac sprue.

### 2.3. Interventions

Each probiotic capsule contained 10 × 10^9^ colony forming units (CFU) of either freeze-dried *B. longum* (Lot Numbers: NH131210-1VB and NH151104-ICP) or *L. paracasei* (Lot Numbers: NH131217-1VB and NH151106-ICP), with potato starch and magnesium stearate as excipients. The placebo (Lot Numbers: NH131226-ISC and NH151028-ICP) contained only potato starch and magnesium stearate. At the baseline visit, participants were instructed to start consuming one capsule of the investigational products with breakfast on the following day (Day 1), and then daily for 8 weeks. The investigational products and placebo were manufactured by Lallemand Health Solutions (LHS) and kept refrigerated (2–8 °C) before use at the study site.

### 2.4. Randomization and Blinding

Eligible participants were assigned a randomization code from a list generated by www.randomization.com, and allocated to each intervention group in a 1:1:1 ratio. Except for 13 participants enrolled while the study was focused on IBS-C, IBS subtypes were not considered before randomization after the eligibility criteria were extended to include all subtypes. The investigational products were labelled in accordance with the International Conference on Harmonisation Good Clinical Practice guidelines, applicable local regulatory guidelines, and included the applicable randomization number. All capsules were identical in size, shape, colour, and taste. Investigators, clinic staff and participants remained blinded for the duration of the study.

### 2.5. Assessments of IBS Symptom Severity, General Health and Psychological Well-Being

The primary outcome measure was the difference in the mean change in symptom severity between each probiotic group and the placebo group (calculated at week 4 and 8, over baseline; i.e., week 4–baseline and week 8–baseline). Symptom severity was assessed using the IBS-SSS.

The secondary outcomes included the difference in the mean change between each probiotic group and the placebo group (calculated at week 4 and 8, over baseline) in several aspects of general health, quality of life, and psychological well-being. The change in general health was assessed using the SF-36 questionnaire; a commonly used tool with 36-items to measure health-related quality of life in 8 domains [20]. Levels of depression and anxiety were measured using the HADS questionnaire; an easy-to-use and validated tool developed for the detection of anxiety disorders and depression in a non-psychiatric hospital clinic setting [21].

Other secondary outcomes were the difference in mean change between each probiotic group and placebo group (calculated at week 4 and 8, over baseline) for each of the following parameters: abdominal pain intensity, abdominal pain frequency, abdominal distension/tightness, bowel habit satisfaction, and IBS-SSS score for each IBS subtypes. Changes in the microbiome composition from baseline to week 8 for each probiotic group and the placebo group were also investigated. Additional secondary outcomes examined were the severity of straining and stool consistency according to the IBS daily diary and amount of rescue medication (Bisacodyl 5 mg, a laxative) used throughout the study for the relief of severe constipation (72 h since last BM or when symptoms of constipation became intolerable).

### 2.6. Fecal Microbe Composition Analysis

Stool samples collected at baseline and week 8 were used to confirm participant compliance and microbe composition through quantitative real-time polymerase chain reaction (qPCR). Total DNA from 250–350 mg of homogenized fecal samples was extracted using the QIAamp Fast DNA Stool Mini kit (QIAGEN, ON, Canada) with the following modifications: (1) two washes with 0.05 M phosphate buffer prior to the InhibitEx step and (2) a 0.1 mm zirconia/silica bead beating step (3 × 4 m/s for 1 min) prior to the centrifugation of samples to pellet particles. Ratios of A260/A280 were assessed to determine sample purity. Absolute quantification of the two probiotic strains and *Bifidobacterium* genus and relative quantification of *Akkermansia mucinphilia* and *Faecalibaterium prausnitzii* was conducted. Template DNA generated for the standard curve formation involved spiking 10^9^ of the appropriate lyophilized powder into a fecal mix and proceeding with the DNA extraction. DNA was diluted 10-fold to generate a standard curve ranging from 10^9^–10^4^ bacteria for *L. paracasei* and *B. longum* Primers for *B. longum* R0175 forward: 5’-GTCGCCACATTTCATCGCAA-3’, reverse: 5’-GAGAGCTTCGATTGGCGAAC-3’; *L. paracasei* HA-196 forward: 5’-ACCGAAGTCTATCACCCGGA-3’, reverse: 5’-TCGCCAAATTTGCTGTCGTG-3’; *Bifidobacterium* genus forward: 5’-TGGAAGGTCTCGATGGAGGT-3’, reverse: 5’-CTGGACAAGCCGTTCCTGAT-3’). DNA was diluted 1/5 prior to qPCR. Each qPCR reaction contained 300 nM of the appropriate forward and reverse primer, 1X SYBR Select Master Mix (Thermo Fisher Scientific, Waltham, MA, USA) and 1 µL of diluted DNA. The epMotion 5075 liquid handling robot (Eppendorf, Hamburg, Germany) was used to dilute DNA from the fecal samples 5-fold, and add 9 mL of mastermix and 1 µL of diluted DNA to the reaction plate. The cycling conditions for the *B. longum* R0175, *L. paracasei* HA-196, *Bifidobacterium* species, and 16S rRNA universal bacterial primers included a 2-min hold at 50 °C, a 2-min hold at 95 °C and 40 cycles of 95 °C for 15 s, 60 °C for 30 s and 72 °C for 30 s. The relative quantifications of *A. muciniphila* and *F. prausnitzii* was performed via species-specific primers and DNA was normalized by bacterial primers targeting the 16S rRNA gene. The primers and cycling conditions used for *A. muciniphila* and *F. prausnitzii* quantification were obtained from literature [22,23,24]. The CFX384™ Touch Real-Time polymerase chain reaction (PCR) Detection System was used to perform quantitative PCR (qPCR) analyses and results were viewed using the CFX Maestro Software 1.1 (Bio-Rad, Hercules, CA).

### 2.7. Compliance

Compliance was assessed by counting the returned IP at the final study visit. Percent compliance was calculated by determining the number of capsules consumed divided by the number expected to have been taken multiplied by 100. Participants were also asked every day in the IBS daily diary if they consumed the IP. In the event of a discrepancy between the information in the IBS daily diary and the amount of IP returned, compliance was determined based on the product returned unless an explanation for the loss of product was provided.

### 2.8. Statistical Analyses

The proposed sample size for this study was 285 enrolled participants, with 95 participants randomized into each of the three study arms in a double-blinded manner. The sample size calculation was based on a standard deviation (SD) of 60, a significance level of 5% (two-sided α), 83% power (β = 0.17), 20% attrition rate and a 110-point detectable difference in IBS-SSS scores from baseline to week 8, compared between probiotics and placebo. The assumptions were based on two previous trials, by Dapoigny et al. (2012) [25] and Williams et al. (2009) [26], which examined the effect of probiotic interventions with *Lactobacillus* and *Bifidobacterium* strains on the symptom severity scores in adult IBS patients.

All statistical analyses were completed using SAS^®^ software Version 9.3 (SAS Institute, Cary, NC, USA) for Microsoft Windows, where probabilities ≤0.05 were considered statistically significant. A blinded interim analysis was conducted on the primary outcome (IBS-SSS) after 50% recruitment. The blind was maintained for all study personnel including the statistician. The per protocol (PP) population consisted of all participants who consumed at least 80% of either product dose, did not have any major protocol violations, and completed all study visits and procedures connected with measurement of the primary variable. The safety population consisted of all participants who received either product and on whom any post-randomization safety information was available. An efficacy analysis based on the PP population and IBS subtypes was performed. Variables were tested for normality and log-normality, and variables showing a log-normal distribution were analyzed in the logarithmic domain.

Appropriate non-parametric tests were used to analyze non-normal variables. Continuous, normally distributed variables were analyzed by linear mixed-effects models with visit (baseline, week 4, and week 8), intervention (*L. paracasei*, *B. longum,* and placebo groups), and their interaction as fixed effects, and subject as a random effect to capture the repeated observations on each subject. Additionally, within-group changes from Day 0 were obtained from the linear mixed-effects models.

## 3. Results

### 3.1. Study Participant Dispositions

**Figure 2:**
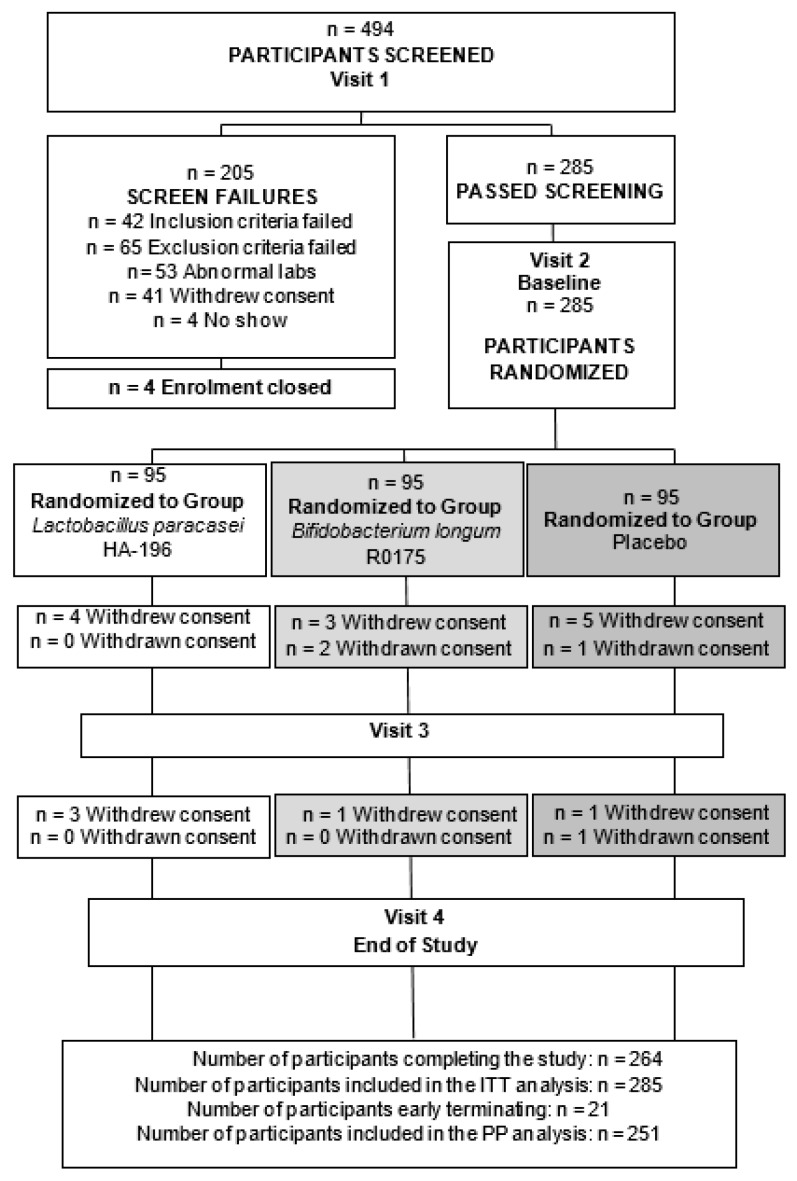
Participant disposition throughout the study. At the end of the study, 251 participants were included in the per protocol population. ITT, intent-to-treat; PP, per protocol.

Out of the 494 participants screened, 285 were eligible and enrolled in the study. Ninety-five participants were randomized into each of the three groups to receive either *L. paracasei*, *B. longum*, or placebo (Figure 2). Following randomization, six participants withdrew consent and 1 participant was lost to follow up in the *L. paracasei* group. In the *B. longum* group, two participants withdrew consent, two were withdrawn by the Qualified Investigator (QI) and two were lost to follow up. In the placebo group, eight participants discontinued the study (three withdrew consent, two were withdrawn by the QI and three were lost to follow up). Thirteen participants who completed the study were removed from per protocol (PP) analysis. Four participants were removed from the *L. paracasei* group due to consumption of magnesium throughout the study (*n* = 1), use of antibiotics (*n* = 1), or insufficient compliance (*n* = 2). Three participants were excluded from the *B. longum* group for consumption of NSAIDs (*n* = 1), antibiotics (*n* = 1), and laxatives (*n* = 1). In the placebo group, six participants were removed from PP analysis due to the consumption of NSAIDs and PPIs (*n* = 1) or antibiotics (*n* = 4), while another was enrolled 18 days outside of the screening window (*n* = 1). The demographic information of the PP population is presented in Table 1, along with the IBS subtype distribution within each treatment conditions.

### 3.2. Improvement in the IBS Symptom Severity Score and Rescue Medication Use

**Figure 3:**
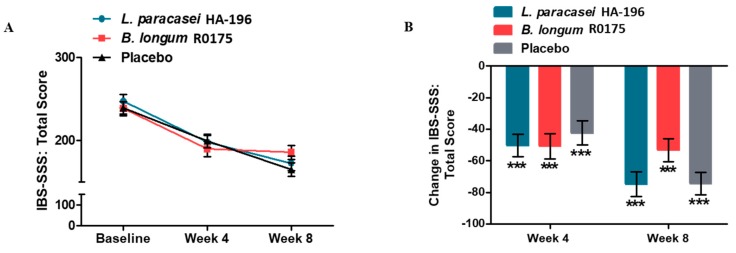
Effect of *Lactobacillus paracasei* HA-196 and *Bifidobacterium longum* R0175 on the IBS symptom severity score. (**A**) Mean Total IBS-SSS at baseline, week 4 and week 8; and (**B**) Mean change in IBS-SSS from baseline to week 4 and week 8 for participants in the PP population. *L. paracasei* group *n* = 84, *B. longum* group *n* = 86, placebo group *n* = 81; data represented as mean ± SD; *** *p* < 0.001; IBS-SSS, irritable bowel syndrome symptom severity scale. *L. paracasei*, *Lactobacillus paracasei* HA-196; *B. longum*, *Bifidobacterium longum* R0175.

**Figure 4:**
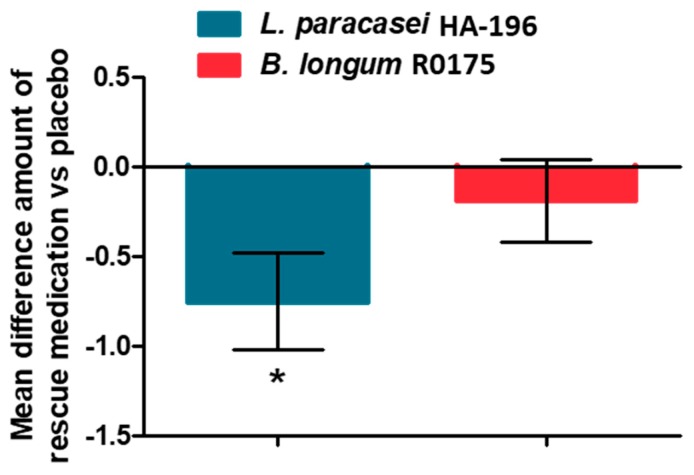
Effect of *Lactobacillus paracasei* HA-196 and *Bifidobacterium longum* R0175 on the use of rescue medication (bisacodyl 5 mg tablets) in participants with IBS compared to placebo. *L. paracasei* group *n* = 84, *B. longum* group *n* = 86, placebo group *n* = 81; data represented as mean ± SD; * *p* < 0.05. *L. paracasei, Lactobacillus paracasei* HA-196; *B. longum*, *Bifidobacterium longum* R0175.

There were no significant between group changes; however, there were significant reductions in IBS-SSS at week 4 and 8 week from baseline in all three groups. The IBS-SSS scores were reduced significantly at week 4 vs. baseline in participants supplemented with *L. paracasei* (−20%), *B. longum* (−20%), and placebo (−17%) (all *p* < 0.001; Table 2 and Figure 3). Similarly, IBS-SSS scores were significantly reduced at week 8 vs. baseline in the *L. paracasei* (−30%), *B. longum* (−22%), and placebo (−31%) groups (all *p* < 0.001; Table 2 and Figure 3). Rescue medication (bisacodyl 5 mg tablets) was permitted for the relief of severe constipation, defined as 72 h since last BM or when symptoms became intolerable. Participants from both probiotic supplemented groups reported consuming less rescue medication compared to the placebo group; this difference was found to be statistically significant only for the *L. paracasei* group (*p* < 0.05; Figure 4, Supplementary Table S2).

### 3.3. Increase in Bowel Movement Frequency in Participants with IBS-C

**Figure 5:**
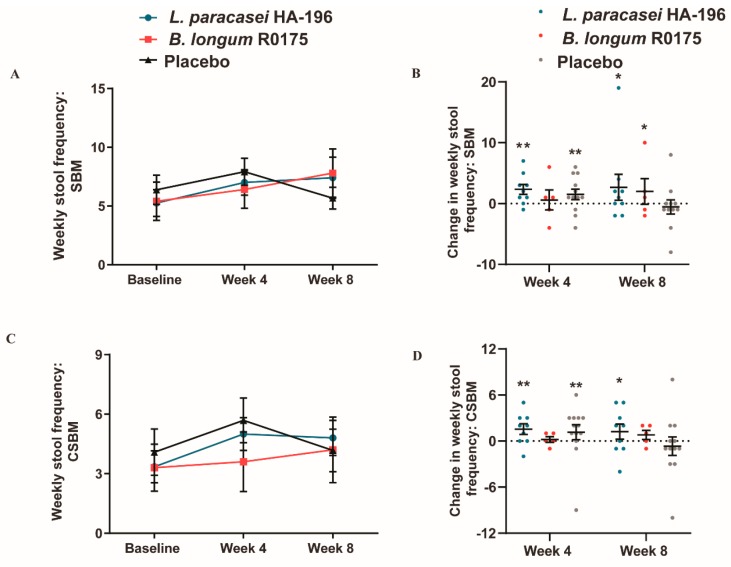
Effect of *Lactobacillus paracasei* HA-196 and *Bifidobacterium longum* R0175 on frequency of bowel movements in participants with IBS-C. (**A**) Weekly SBM at baseline, week 4 and week 8, (**B**) Change in total SBM from baseline to week 4 and week, (**C**) Total CSBM at baseline, week 4 and week 8, and (**D**) Change in weekly CSBM from baseline to week 4 and week 8. *L. paracasei* group *n* = 9, *B. longum* group *n* = 5, placebo group *n* = 13; data represented as mean ± SD; * *p* < 0.05, ** *p* < 0.01; SBM; spontaneous bowel movement; CSBM, complete spontaneous bowel movement; *L. paracasei*, *Lactobacillus paracasei* HA-196; *B. longum, Bifidobacterium longum* R0175.

In participants with IBS-C, there were no significant between-group differences in SBM, CSBM, and stool consistency among the three groups (Table 3). However, *L. paracasei* and *B. longum* consumption led to increased number of within-group SBM and CSBM. At week 4, participants with IBS-C reported significant 33% and 24% improvements for the number of weekly SBM from baseline when consuming either *L. paracasei* or placebo, respectively (both *p* = 0.007). Although, participants receiving *B. longum* reported a 19% increase in SBM, it was not statistically significant until week 8, when a 44% improvement was reported (*p* = 0.03). At week 8, participants with IBS-C consuming *L. paracasei* continued to report a significant 33% improvement in the number of weekly SBM from baseline (*p* = 0.03). Conversely, a non-significant 11% decrease in the number of weekly SBM from baseline was reported by the placebo group (Figure 5A,B).

IBS-C participants consuming *L. paracasei* reported a significant 49% increase in CSBM at week 4, and a significant 43% increase at week 8 (*p* = 0.004 and *p* = 0.02, respectively). Whereas, the placebo group had reported a significant 39% increase (*p* = 0.04) at week 4 in the number of CSBM, but by the end of the study period a non-significant 2.2% decrease was reported from baseline (Figure 5C,D).

### 3.4. Decrease in the Bowel Movement Frequency in Participants with IBS-D

**Figure 6:**
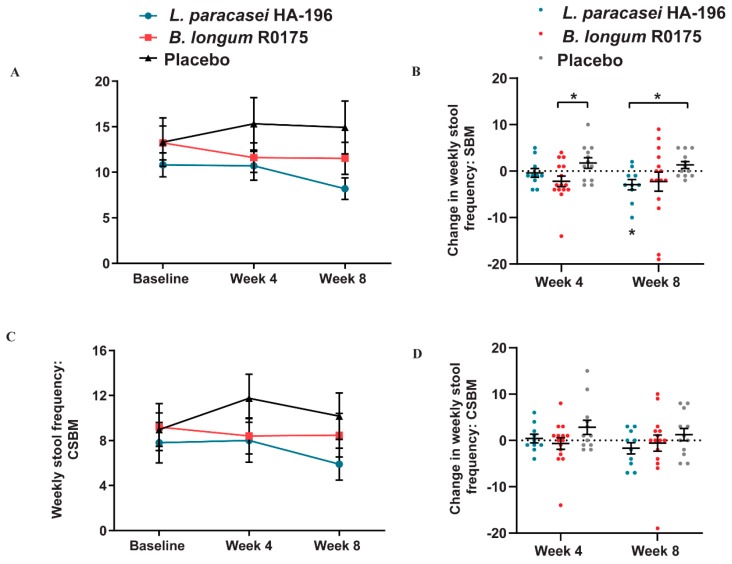
Effect of *Lactobacillus paracasei* HA-196 and *Bifidobacterium longum* R0175 on frequency of bowel movements in participants with IBS-D. (**A**) Weekly SBM at baseline, week 4 and week 8, (**B**) Change in total SBM from baseline to week 4 and week 8, (**C**) Total CSBM at baseline, week 4 and week 8, and (**D**) Change in weekly CSBM from baseline to week 4 and week 8. *L. paracasei* group *n* = 10, *B. longum* group *n* = 15, placebo group *n* = 12; data represented as mean ± SD; * *p* < 0.05, ** *p* < 0.01; SBM; spontaneous bowel movement; CSBM, complete spontaneous bowel movement; *L. paracasei*, *Lactobacillus paracasei* HA-196; *B. longum, Bifidobacterium longum* R0175.

In participants with IBS-D, *L. paracasei* and *B. longum* consumption led to lower within group SBM and CSBM frequencies. The *L. paracasei* group reported a significant 21% decrease in SBM from baseline to week 8 that was decreased compared to placebo at week 8 (*p* < 0.05) (Figure 6A,B). Additionally, IBS-D participants supplemented with *B. longum* reported significant decreases in their SBM stool frequency compared to placebo at week 4 (Figure 6B). With respect to CSBM, there were no significant within-group differences in participants with IBS-D. The greatest reduction, however, in CSBM was reported by participants receiving *L. paracasei* (24%), followed by the *B. longum* group (8%); whereas participants in the placebo group reported a 13.5% increase from baseline to week 8 (Figure 6C,D).

Furthermore, IBS-D participants in the *L. paracasei* and placebo groups reported significant decreases in stool consistency at 4 weeks; however, the *L. paracasei* group was the only group to report a significant 15% within group decrease in stool consistency after 8 weeks (Table 4). There were no significant between-group differences for CSBM or stool consistency among the three groups at the end of 4 or 8 weeks of the supplementation period.

### 3.5. No Significant Changes in the Bowel Movement Frequency in Participants with IBS-M

**Figure 7:**
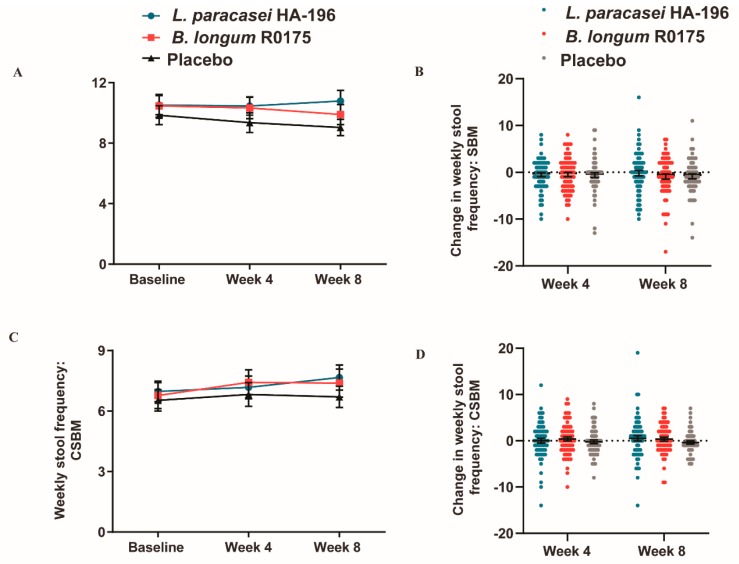
Effect of *Lactobacillus paracasei* HA-196 and *Bifidobacterium longum* R0175 on frequency of bowel movements in participants with IBS-M. (**A**) Weekly SBM at baseline, week 4 and week 8, (**B**) Change in total SBM from baseline to week 4 and week 8, (**C**) Total CSBM at baseline, week 4 and week 8, and (**D**) Change in weekly CSBM from baseline to week 4 and week 8. *L. paracasei* group *n* = 64, *B. longum* group *n* = 66, placebo group *n* = 55; data represented as mean ± SD; * *p* < 0.05, ** *p* < 0.01; SBM; spontaneous bowel movement; CSBM, complete spontaneous bowel movement; *L. paracasei, Lactobacillus paracasei* HA-196; *B. longum*, *Bifidobacterium longum* R0175.

In participants with IBS-M, *L. paracasei* or *B. longum* consumption did not result in any significant between or within group differences in SBM or CSBM. The *L. paracasei* group reported a 2.6% increase, whereas the *B. longum* and placebo groups reported 5.5% and 8.1% decreases, respectively, in SBM from baseline to week 8 (Figure 7A,B). At week 8, participants consuming *L. paracasei* and *B. longum* reported 9.9% and 9.0% increases in CSBM, respectively, while those on placebo reported a 2.6% decrease (Figure 7C,D). All groups reported decreases in stool consistency from baseline to week 4, however, the only significant decrease of 6.6% was reported by the placebo group (*p* = 0.025). There were, however, no significant between-group differences in stool consistency among the three groups (data not shown).

### 3.6. Improvements in Mental Health Measures in Participants Given Probiotics

**Figure 8:**
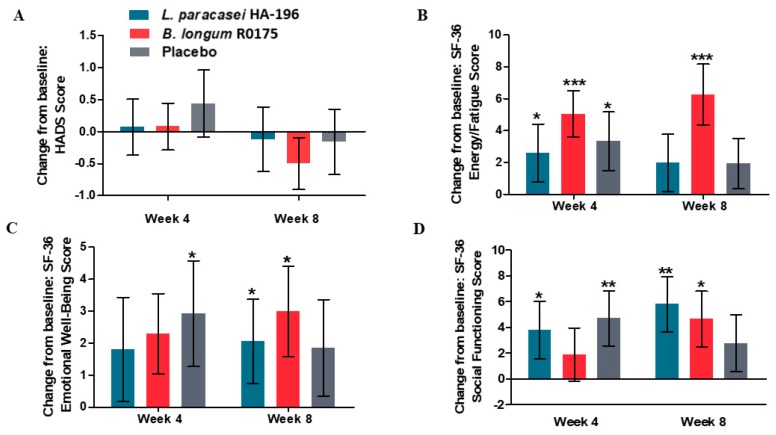
Effect of *Lactobacillus paracasei* HA-196 and *Bifidobacterium longum* R0175 on psychological symptoms in participants with IBS from baseline to week 4 and week 8 (**A**) Mean change from baseline in HADS score, (**B**) Mean change from baseline in SF-36 energy levels, (**C**) Mean change from baseline in SF-36 emotional well-being, and (**D**) Mean change from baseline in SF-36 social functioning. *L. paracasei* group *n* = 84, *B. longum* group *n* = 86, placebo group *n* = 81; data represented as mean ± SD; * *p* < 0.05, ** *p* < 0.01, *** *p* < 0.001; HADS, Hospital Anxiety and Depression Scale; SF, Short Form; *L. paracasei, Lactobacillus paracasei* HA-196; *B. longum*, *Bifidobacterium longum* R0175.

There were no significant within group differences in HADS score, but participants receiving *B. longum* reported the greatest reduction (4%, *p* = 0.09) in HADS score after 8 weeks (Figure 8A). As assessed by the SF-36, participants supplemented with *B. longum* reported a significant increase of 12% (*p* < 0.001) and those in the *L. paracasei* group reported a non-significant 3.7% increase in energy from baseline to week 8 (Figure 8B). Evaluation of the emotional well-being parameter by SF-36 showed, significant 3% and 4% improvements in the *L. paracasei* and *B. longum* groups, respectively, from baseline to week 8 (all *p* < 0.05, Figure 8C). Participants receiving *L. paracasei* and *B. longum* reported significant 8% and 6% within-group increases in social functioning, respectively (all *p* < 0.05, Figure 8D). These within-group significant improvements in SF-36 parameters were not reported by participants given placebo. There were also no significant between-group differences among the three groups in the SF-36 parameters assessed.

### 3.7. L. paracasei and B. longum are Safe to Consume

A total of 89 adverse events (AEs) were reported by 72 participants in this study. All AEs, except two (ankle sprain and intestinal prolapse) were resolved by the end of the study and not related to the IPs. There we no significant differences between the probiotic groups and placebo group in number of participants reporting AEs. A single serious AE was reported (miscarriage), which was assessed as unrelated to IPs by the Investigator and self-resolved by the end of the study. All safety parameters were within clinically acceptable ranges and both probiotic products were considered safe.

### 3.8. High Compliance and Increased Number of Participants with Bifidobacterium Species

**Figure 9:**
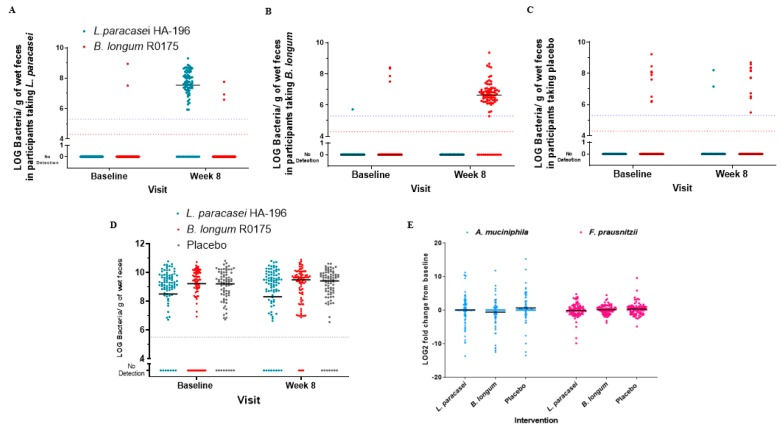
Effect of *Lactobacillus paracasei* HA-196 and *Bifidobacterium longum* R0175 on fecal microbial composition in participants with IBS. qPCR analysis of absolute quantification of *B. longum* (red dots) and *L. paracasei* (blue dots) in participants receiving (**A**) *L. paracasei* (**B**) *B. longum, or* (**C**) placebo; (**D**) Absolute quantification of *Bifidobacterium* species at baseline and 8 weeks with limit of quantification for each species represented by corresponding coloured dotted line; (**E**) relative quantification of *Akkermansia muciniphila* and *Faecalibacterium prausnitzii* in the three groups. *A. muciniphila*, *Akkermansia muciniphila*; *F. prausnitzii*, *Faecalibacterium prausnitzii*; *B. longum*, *Bifidobacterium longum*; *L. paracasei*, *Lactobacillus paracasei*.

In addition to assessing compliance based on counting capsules at each study visit, qPCR analysis was used to quantify probiotic strains in fecal samples and establish participant compliance. Compliance was found to be of 85% and 83% for participants receiving *B. longum* and *L. paracasei*, respectively. Probiotic strains were detected in their respective groups and in nine and eleven participants of the placebo group at baseline and the end of study, respectively (Figure 9A–C). We observed no significant changes in fecal *Bifidobacterium* species levels at baseline and at the end of study (median ~9 Log bacteria/g of wet feces). Notably, following *B. longum* administration, the number of participants without detection of *Bifidobacterium* species was only 4 participants at the end of the supplementation period (Figure 9D). There were no significant within group changes in relative quantification of *A. muciniphila* and *F. prausnitzii* in any of the study groups (Figure 9E). There were no differences in *L. paracasei*, *B. longum* or *Bifidobacterium* species levels in participants with IBS-C, -D or -M after supplementation (Supplementary Figure S1).

## 4. Discussion

In this randomized, double-blind, placebo-controlled study, both *L. paracasei* HA-196 and *B. longum* R0175 were found to be safe, and significantly improved participants’ experience of IBS-related symptoms after 8 weeks as reflected by reduced scores on the IBS-SSS. A similar decrease in symptom severity was also observed in the placebo group. The significant reduction in symptom severity was marked by a 30% decrease in *L. paracasei* and a 22% decrease in the *B. longum* groups compared to a 31% decrease in the placebo group. IBS-SSS is a widely used self-administered questionnaire that measures severity and frequency of abdominal pain, abdominal distension/tightness, bowel habit satisfaction, and quality of life. The total IBS-SSS score ranges from 0 to 500, with higher scores indicating more severe symptoms. A decrease in the IBS-SSS score indicates a reduction in symptom severity. When participants were grouped into IBS subtypes (IBS-C, IBS-D, IBS-M), a reduction in IBS-SSS was also observed in all three groups. However, *L. paracasei* supplemented IBS-C and IBS-D participants reported improvements in their bowel habits and stool consistency that were not observed in the placebo group.

In addition to IBS-SSS score, BM frequency was examined based on IBS subtype. At the end of the 8-week study, IBS-C participants in the *L. paracasei* group had significant improvements in both SBM and CSBM. Although not statistically significant, IBS-C participants in the placebo group reported fewer numbers of SBM and CSBM at the end of the study. For IBS-D participants, *B. longum* significantly reduced SBM compared to placebo from baseline to week 4, but this significant between-group difference was not sustained to the end of study. *L. paracasei* significantly improved the weekly frequency of SBM at week 8 in IBS-D participants compared to the placebo. Additionally, *L. paracasei* had a greater effect on reducing the number of CSBM in IBS-D participants than *B. longum*. As with IBS-C participants, the placebo did not alter bowel movement frequency with IBS-D. Although, not significantly different, *L. paracasei* supplemented IBS-M participants were the only group to report increases in SBM and CSBM after 8 weeks. IBS-M participants in the *B. longum* group did not report increases in SBM in participants, and those in the placebo had decreases in CSBM by the end of the study.

All participants with IBS who received *L. paracasei* reported improvements in stool consistency after 8 weeks, unlike IBS-C and IBS-M participants in the *B. longum* group as well as all participants given placebo. This shift in stool consistency in the *L. paracasei* groups was marked by a significant within-group reduction in stool consistency reported by IBS-D participants after 8 weeks. The monitoring of stool consistency and BM frequencies provide evidence of efficacy of the two probiotic strains, with *L. paracasei* being more efficacious in improving symptoms of IBS, particularly IBS-C and IBS-D.

An important aspect of this study was that of all IBS subtypes, the highest population of participants enrolled was those with IBS-M. IBS as a condition can be very transient in presentation of its type and intensity [27]. Participants in this study often changed their subtype between screening and baseline run-in. Furthermore, the recording of IBS intensity is highly subjective making clinical studies in this area challenging. Despite the challenges associated with studies in this area, this study suggests that both *L. paracasei* HA-196 and *B. longum* R0175 play a role in mitigating the effects of IBS in certain subtypes. These data would suggest that probiotic efficacy may be dependent on the IBS subtype. This warrants further investigation and studies focused on specific IBS symptoms within the IBS symptomatology continuum rather than considering all IBS patients as a homogenous population.

Psychological comorbidities are commonly observed in IBS patients, who are significantly more likely to develop mental health conditions such as depressive disorders, anxiety, and sleep disorders [28]. Considering this, probiotics consumption is viewed as a means to alleviate the psychological comorbidities of IBS [29]. However, similar to the efficacy of probiotics and the physical symptoms of IBS, there is heterogeneity among studies linking probiotics and modulation of behaviour in humans. A narrative review of seven systematic reviews of RCTs in individuals with depressive symptoms noted that five reviews reported a beneficial effect of probiotics on depressive symptoms. However, the authors stated that heterogeneity in the type of populations studied warranted further studies in clinically diagnosed depression before more definitive conclusions could be made [30]. More recently, a study on IBS participants with mild to moderate anxiety and/or depression reported that *B. longum* NCC3001 improved depression scores based on the HADS scale. A reduction of two or more points was reported in 64% of the participants in the probiotic group compared to 32% in the placebo group (*p* = 0.04) [31], although anxiety or IBS symptoms scores were not different compared to placebo. In the current study, we did not observe any significant improvements in the HADS score nor in general health assessment (based on the SF-36) among IBS participants compared to placebo. However, participants in the *B. longum* group reported having increased energy levels after 8 weeks, which can directly impact their willingness and ability to perform everyday tasks. Furthermore, participants in both *B. longum* and *L. paracasei* groups reported significant within-group improvements in social functioning after 8 weeks. In contrast, none of these improvements pertaining to mental health were found in participants receiving the placebo.

Ensuring compliance with the probiotic for the duration of the study is an essential component of examining efficacy. The ability to detect the probiotic strains in participant’s fecal samples can confirm the participant compliance calculated from the returned IP. Fecal samples from baseline and 8 weeks were analyzed by qPCR and revealed a high degree of compliance within the probiotic supplemented groups. In the placebo group, probiotic strain detection was not expected. However, a handful of participants in the placebo group did have detection, which may potentially be due to the participants harbouring similar sequences from the bacteria in their gut, which may occur naturally due to bacterial diversity.

The abundance of selected relevant bacteria, including *A. muciniphila*, *F. prausnitzii* and *Bifidobacterium* species was also investigated in fecal samples by qPCR. Compared to baseline, participants in all three treatment groups had no significant changes in the two species and one genus quantified, though more participants had detection of *Bifidobacterium* species in their fecal matter following 8-weeks of *B. longum* supplementation. The latter may have contributed to the BM improvement observed in the *B. longum* supplemented groups, as *Bifidobacterium* species have been shown to improve bowel habits in all IBS subtypes [32]. An increase in *A. muciniphila* has been associated with a decrease in abdominal pain in IBS patients, and *F. prausnitzii* has been shown to reduce visceral hypersensitivity in a non-inflammatory animal model of IBS [33,34]. Thus, it is possible that the unchanged relative abundance of *A. muciniphila* and *F. prausnitzii* following probiotic supplementation may have contributed to the absence of significant differences in the IBS-SSS score between the groups. It also suggests that the potential mechanism by which *L. paracasei*, exerted its impact on bowel habits and stool consistency may be independent of the specific bacterial species investigated.

A placebo effect of 33% was observed in this study, consistent with reports from previous IBS clinical trials. A review of RCTs evaluating complementary health products showed an overall placebo response of 43%, with higher placebo responses associated with longer treatment duration and greater number of clinic visits [35]. A meta-analysis reported that the placebo response ranges from 16% to 71% in participants with IBS, with a population-weighted average of 40% in studies using natural health products and pharmaceutical interventions [36]. The subjective nature of the assessments and the intrinsic nature of IBS patients who display high levels of suggestibility and a strong placebo effect may have contributed to the placebo effect seen in this study. Additionally, this placebo effect may partially explain why some outcomes assessed were significant at either week 4 or 8. The placebo effect in studies within this area of research also exemplifies the importance of a run-in period, which was included in this study. However, examining bowel habits for a longer period prior to intervening may be an important aspect of understanding probiotic efficacy while mitigating the placebo effect.

Other limitations of this study include the relatively small sample size of the population, more specifically when considering the IBS subtypes, which may have introduced variability in the responses and reduced effect sizes. Moreover, the severity of IBS symptoms using IBS-SSS was only captured at baseline, week 4, and week 8 and not at any of the intermediate weeks. A characteristic feature of IBS is that some weeks were worse than the others, and due to the lack of data from intermediate weeks, temporary improvements experienced by participants associated with probiotic supplementation may have been missed. Furthermore, the duration of IBS in participants prior to enrollment into the study was not accounted for, which may have influenced the outcomes measured. Additionally, participants in this study were diagnosed and classified by IBS subtype according to Rome III, which has now been updated to Rome IV [37]. A recent study has shown differences in the diagnosis and classification of participants with IBS based on Rome III or Rome IV [38].Of note, at the time of classification based on the Rome III questionnaire, no participants in this study were classified as the IBS-unclassified (IBS-U) subtype.

Low grade inflammation has been suggested to play a role in the pathogenesis of IBS. Abnormal mast cell activation in the gut has been associated with the development of IBS symptoms [39]. Although beyond the scope of this study, it would be important to evaluate the anti-inflammatory effect of *L. paracasei* or *B. longum* in IBS patients. Overall, the results of the current study suggest a clinically relevant role for *L. paracasei* or *B. longum* as probiotics to reduce symptoms in IBS. Furthermore, they may add to the armamentarium of IBS management tools without the safety concerns that may accompany pharmaceuticals. The effects of each probiotic strain may be dependent on the IBS subtype, and future studies should target IBS-D and IBS-M for probiotic therapy.

## Figures and Tables

**Table 1:** Demographic characteristics for all participants in the per protocol (PP) population and their IBS-subtypes.

Parameter	*L. paracasei* HA-196 (*n*)	*B. longum* R0175 (*n*)	Placebo (*n*)
**Age (years)**			
Mean+/−SD	42.42 ± 12.30 (84)	42.31 ± 16.88 (86)	41.84 ± 16.14 (81)
Median (Min–Max)	43.00 (21.00–72.00)	41.00 (19.00–87.00)	40.00 (18.00–75.00)
*p*-value *	0.9573	0.9706	
**Gender [*n* (%)]**			
Female	67 (79.8%)	64 (74.4%)	64 (79.0%)
Male	17 (20.2%)	22 (25.6%)	17 (21.0%)
*p*-value *	1.0000	0.5836	
**Ethnicity [*n* (%)]**			
Hispanic or Latino	7 (8.3%)	5 (5.8%)	1 (1.2%)
Not Hispanic or Latino	77 (91.7%)	81 (94.2%)	80 (98.8%)
*p*-value *	0.0641	0.2115	
**Race [*n* (%)]**			
Black or African American		2 (2.3%)	2 (2.5%)
Central American	1 (1.2%)	1 (1.2%)	
East Asian			2 (2.5%)
Eastern European White	10 (11.9%)	9 (10.5%)	8 (9.9%)
Middle Eastern	8 (9.5%)	2 (2.3%)	3 (3.7%)
North American Indian		1 (1.2%)	1 (1.2%)
South American	5 (6.0%)	3 (3.5%)	1 (1.2%)
South Asian		2 (2.3%)	1 (1.2%)
South East Asian	1 (1.2%)		1 (1.2%)
Western European White	59 (70.2%)	66 (76.7%)	62 (76.5%)
*p*-value *	0.1463	0.8926	
**Type of IBS: ROME III [*n* (%)]**			
IBS-C	10 (11.9%)	5 (5.8%)	13 (16.0%)
IBS-D	10 (11.9%)	15 (17.4%)	13 (16.0%)
IBS-M	64 (76.2%)	66 (76.7%)	55 (67.9%)
*p*-value *	0.5434	0.1041	

IBS, irritable bowel syndrome; IBS-C, constipation-predominant IBS, IBS-D, diarrhea-predominant IBS; IBS-M, mixed bowel habits IBS; *n*, number; SD, standard deviation; Min, minimum; Max, maximum; * Continuous variables: *p*-values for comparison of each probiotic strain to placebo generated by ANOVA with Group as a fixed effect and subject as a random effect. Dunnett’s method was used to adjust for multiple comparisons; * Categorical variables: *p*-values for comparison of each probiotic strain to placebo generated by Fisher’s Exact (2-tail) test.

**Table 2:** Irritable Bowel Syndrome Symptom Severity Scale (IBS-SSS) score at baseline, week 4, and week 8 and change in IBS-SSS from baseline to week 4, and week 8 for participants in the PP population.

IBS-SSS				
Study Day	Statistic	*L. paracasei* HA-196	*B. longum* R0175	Placebo
Baseline (Day 0)	Mean ± SD (*n*)	246.94 ± 75.53 (84)	238.13 ± 76.59 (83)	238.96 ± 64.55 (81)
	*p*-value *	0.7011	0.9960	
Week 4	Mean ± SD (*n*)	198.22 ± 69.07 (82)	189.57 ± 82.55 (83)	199.39 ± 72.13 (77)
Week 8	Mean ± SD (*n*)	172.23 ± 80.90 (84)	185.71 ± 77.74 (84)	164.56 ± 73.25 (81)
Change from Baseline to Week 4	Mean ± SD (*n*)	−50.20 ± 64.42 (82)	−50.78 ± 72.12 (82)	−42.32 ± 66.93 (77)
	Within Group *p*-Value +	*p* < 0.001 (r)	*p* < 0.001 (r)	*p* < 0.001 (r)
	Between Group *p*-value **	0.4246 (r)	0.1390 (r)	
Change from Baseline to Week 8	Mean ± SD (*n*)	−74.71 ± 71.52 (84)	−53.25 ± 65.65 (83)	−74.41 ± 62.83 (81)
	Within Group *p*-Value +	*p* < 0.001 (r)	*p* < 0.001 (r)	*p* < 0.001 (r)
	Between Group *p*-value **	0.9632 (r)	0.0763 (r)	

*n*, number; SD, standard deviation; Min, minimum; Max, maximum; * For Baseline (Day 0), *p*-values for comparison of each probiotic strain to placebo generated by ANOVA with Group as a fixed effect and subject as a random effect. Dunnett’s method was used to adjust for multiple comparisons; ** Values generated from a Repeated Measures ANCOVA with baseline as a covariate and Group, Study Day and Group by Study Day interaction as fixed effects with subject as a random effect. *p*-values for comparison to placebo were adjusted for multiple comparisons using Dunnett’s method; + Within group *p*-values generated from the Repeated Measures ANCOVA specified above; (r) indicates values were ranked prior to generating ANOVA or ANCOVA.

**Table 3:** Stool consistency at baseline, week 4, and week 8 and change in stool consistency from baseline to week 4, and week for participants with IBS-C in the PP population.

Study Day	Statistic	*L. paracasei* HA-196	*B. longum* R0175	Placebo
Baseline (average of Weeks −2 and −1)	Mean ± SD (*n*)	2.84 ± 1.43 (10)	2.55 ± 1.57 (5)	2.69 ± 0.78 (12)
	*p*-value *	0.9467	0.9656	
Week 4	Mean ± SD (*n*)	3.14 ± 1.05 (10)	3.21 ± 1.15 (5)	2.91 ± 0.82 (13)
Week 8	Mean ± SD (*n*)	3.21 ± 1.32 (10)	2.44 ± 0.74 (5)	2.49 ± 0.95 (12)
Change from Baseline to Week 4	Mean ± SD (*n*)	0.44 ± 1.09 (9)	0.49 ± 1.74 (5)	−0.05 ± 0.80 (12)
	Within Group *p*-Value +	*p* = 0.221 (r)	*p* = 0.210 (r)	*p* = 0.295 (r)
	Between Group *p*-value **	0.2585 (r)	0.9940 (r)	
Change from Baseline to Week 8	Mean ± SD (*n*)	0.62 ± 1.12 (9)	−0.28 ± 1.54 (5)	−0.41 ± 1.00 (11)
	Within Group *p*-Value +	*p* = 0.186 (r)	*p* = 0.789 (r)	*p* = 0.858 (r)
	Between Group *p*-value **	0.0721 (r)	0.9805 (r)	

*n*, number; SD, standard deviation; Min, minimum; Max, maximum; * For Baseline (Day 0), *p*-values for comparison of each probiotic strain to placebo generated by ANOVA with Group as a fixed effect and subject as a random effect. Dunnett’s method was used to adjust for multiple comparisons; ** Values generated from a Repeated Measures ANCOVA with baseline as a covariate and Group, Study Day and Group by Study Day interaction as fixed effects with subject as a random effect. *p*-values for comparison to placebo were adjusted for multiple comparisons using Dunnett’s method; + Within group *p*-values generated from the Repeated Measures ANCOVA specified above; (r) indicates values were ranked prior to generating ANOVA or ANCOVA.

**Table 4:** Stool consistency at baseline, week 4, and week 8 and change in stool consistency from baseline to week 4, and week 8 for participants with IBS-D in the PP population.

Study Day	Statistic	*L. paracasei* HA-196	*B. longum* R0175	Placebo
Baseline (average of Weeks −2 and −1)	Mean ± SD (*n*)	4.34 ± 0.65 (10)	4.46 ± 0.89 (15)	4.79 ± 0.77 (13)
	*p*-value *	0.3106	0.4416	
Week 4	Mean ± SD (*n*)	3.73 ± 0.75 (10)	4.13 ± 0.64 (15)	4.26 ± 0.57 (12)
Week 8	Mean ± SD (*n*)	3.68 ± 0.95 (10)	4.22 ± 0.97 (15)	4.66 ± 0.76 (12)
Change from Baseline to Week 4	Mean ± SD (*n*)	−0.39 ± 0.80 (10)	−0.36 ± 1.01 (15)	−0.60 ± 0.74 (12)
	Within Group *p*-Value +	*p* = 0.023	*p* = 0.132	*p* = 0.016
	Between Group *p*-value **	0.8523	0.9868	
Change from Baseline to Week 8	Mean ± SD (*n*)	−0.45 ± 1.14 (10)	−0.26 ± 1.12 (15)	−0.20 ± 0.78 (12)
	Within Group *p*-Value +	*p* = 0.013	*p* = 0.281	*p* = 0.450
	Between Group *p*-value **	0.2896	0.5955	

*n*, number; SD, standard deviation; Min, minimum; Max, maximum; * For Baseline (Day 0), *p*-values for comparison of each probiotic strain to placebo generated by ANOVA with Group as a fixed effect and subject as a random effect. Dunnett’s method was used to adjust for multiple comparisons; ** Values generated from a Repeated Measures ANCOVA with baseline as a covariate and Group, Study Day and Group by Study Day interaction as fixed effects with subject as a random effect. *p*-values for comparison to placebo were adjusted for multiple comparisons using Dunnett’s method; + Within group *p*-values generated from the Repeated Measures ANCOVA specified above; (r) indicates values were ranked prior to generating ANOVA or ANCOVA.

## Supplementary Materials

The following are available online at https://www.mdpi.com/2072-6643/12/4/1159/s1, Table S1: CONSORT 2010 checklist of information to include when reporting a randomised trial. Table S2: Number of rescue medication pills taken by participants with IBS-C, -D or -M during the 8-week intervention period. Figure S1: *L. paracasei*, *B. longum* or *Bifidobacterium* species levels in participants with IBS-C, -D or -M after supplementation.
