# Supplementary material for: Efficacy of Lactobacillus paracasei HA-196 and Bifidobacterium longum R0175 in Alleviating Symptoms of Irritable Bowel Syndrome (IBS): A Randomized, Placebo-Controlled Study
Source: Nutrients. 2020 Apr 21;12(4):1159. doi: 10.3390/nu12041159 (PMC7230591; doi:10.3390/nu12041159)
Supplement: Supplementary file 1 [file nutrients-12-01159-s001.zip › Nutrients 725812 Supplementary Materials.docx]

Supplementary Table S2. Number of rescue medication pills taken by participants with IBS-C, -D or -M during the 8-week intervention period

|  | *L. paracasei* (n) | *B. longum* (n) | Placebo (n) |
| --- | --- | --- | --- |
| IBS-C | 4 (2) | 18 (1) | 23 (4) |
| IBS-D | 6 (1) | 0 (0) | 0 (0) |
| IBS-M | 10 (3) | 18 (6) | 18 (3) |

*n indicates the number of participants in each group who took rescue medication

Total number of participants: *L. paracasei* group - 84, *B. longum* group - 86, placebo group – 81


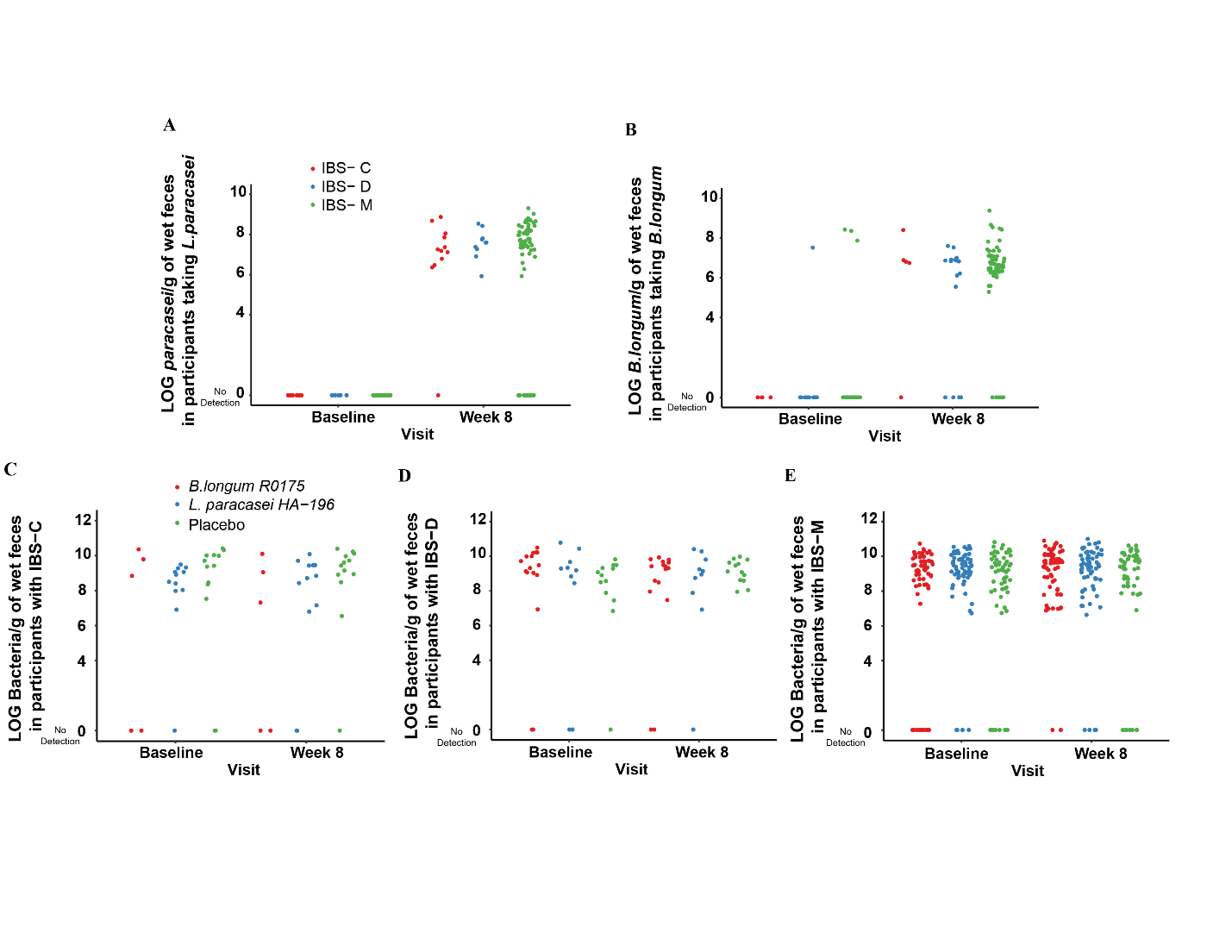
Supplementary Figure S1. *L. paracasei*, *B. longum* or *Bifidobacterium* species levels in participants with IBS-C, -D or – M after supplementation.
